# Individualised ^177^Lu-DOTATATE treatment of neuroendocrine tumours based on kidney dosimetry

**DOI:** 10.1007/s00259-017-3678-4

**Published:** 2017-03-22

**Authors:** Anna Sundlöv, Katarina Sjögreen-Gleisner, Johanna Svensson, Michael Ljungberg, Tomas Olsson, Peter Bernhardt, Jan Tennvall

**Affiliations:** 1 Department of Oncology and Pathology, Clinical Sciences, Lund University, Lund, Sweden; 2 Department of Oncology, Skåne University Hospital, SE-221 85 Lund, Sweden; 3 Department of Medical Radiation Physics, Clinical Sciences, Lund University, Lund, Sweden; 4 Department of Oncology, Sahlgrenska University Hospital, Gothenburg, Sweden; 5 Department of Radiation Physics, University of Gothenburg, Gothenburg, Sweden; 6 Department of Medical Physics and Biomedical Engineering, Sahlgrenska University Hospital, Gothenburg, Sweden

**Keywords:** ^177^Lu-DOTATATE, Neuroendocrine, Dosimetry, PRRT, Renal function

## Abstract

**Purpose:**

To present data from an interim analysis of a Phase II trial designed to determine the feasibility, safety, and efficacy of individualising treatment based on renal dosimetry, by giving as many cycles as possible within a maximum renal biologically effective dose (BED).

**Method:**

Treatment was given with repeated cycles of 7.4 GBq ^177^Lu-DOTATATE at 8-12-week intervals. Detailed dosimetry was performed in all patients after each cycle using a hybrid method (SPECT + planar imaging). All patients received treatment up to a renal BED of 27 ± 2 Gy (α/β = 2.6 Gy) (Step 1). Selected patients were offered further treatment up to a renal BED of 40 ± 2 Gy (Step 2). Renal function was followed by estimation and measurement of the glomerular filtration rate (GFR).

**Results:**

Fifty-one patients were included in the present analysis. Among the patients who received treatment as planned, the median number of cycles in Step 1 was 5 (range 3-7), and for those who completed Step 2 it was 7 (range 5-8); 73% were able to receive >4 cycles. Although GFR decreased in most patients after the completion of treatment, no grade 3-4 toxicity was observed. Patients with a reduced baseline GFR seemed to have an increased risk of GFR decline. Five patients received treatment in Step 2, none of whom exhibited a significant reduction in renal function.

**Conclusions:**

Individualising PRRT using renal dosimetry seems feasible and safe and leads to an increased number of cycles in the majority of patients. The trial will continue as planned.

**Electronic supplementary material:**

The online version of this article (doi:10.1007/s00259-017-3678-4) contains supplementary material, which is available to authorized users.

## Introduction

Data from several clinical trials show peptide-receptor radionuclide therapy (PRRT) to be a well-tolerated and effective treatment of neuroendocrine tumours (NETs) [1–8]. Although PRRT has been used for the treatment of NETs for many years, there is still room for optimisation in some aspects, one of them being individualised treatment taking into account patient-specific factors to maximise efficacy and minimise toxicity. Dosimetry-based treatment is one way of achieving this.

The dose-limiting organs for PRRT are the bone marrow and the kidneys, the latter being a greater problem when using ^90^Y-DOTATOC than with ^177^Lu-DOTATATE [9, 10]. One way of optimising PRRT is to ensure that the tumour receives as many cycles of therapy as possible within reasonable limits for the risk organs. This individualised approach to PRRT has been previously evaluated by Sandstrom *et al* [11], demonstrating a high variability in the number of cycles achieved using this treatment strategy and a renal absorbed dose (AD) limit of 23 Gy. The current protocol is based on the same principle, but aims to explore the limits of bone marrow and renal dose by putting the dosimetric results in relation to clinical effects and using higher dose limits. This individualised approach demands detailed dosimetry which, in the case of ^177^Lu-DOTATATE, can be achieved using post-therapeutic scintigraphy, owing to its characteristic of emitting not only the (therapeutic) β^-^-radiation, but also γ-radiation.

There are no established dose limits for bone marrow and kidneys for ^177^Lu-PRRT, so such data has to be extrapolated from external beam radiotherapy (EBRT) and PRRT with ^90^Y-DOTATOC. Data from external bilateral kidney irradiation indicate a 5% risk of renal dysfunction at 5 years (TD_5/5_) at a mean AD of 18-23 Gy and 0.5-1.25 Gy/fraction [12], or at an AD of 15-18 Gy and 2-Gy fractions [13]. The AD leading to a 50% risk of renal damage at 5 years (TD_50/5_) has been estimated to be 28 Gy [12, 13]. Data from EBRT cannot, however, be directly applied to PRRT due to what can be summarised as the intrinsic differences between external and systemic radiotherapy: different dose rates and fractionation schemes, an inhomogeneous absorbed dose distribution and possibly different radiobiological mechanisms of cytotoxicity resulting in varying biological effect, despite the same amount of energy deposited per unit mass. The linear-quadratic (LQ) radiobiological model is used to convert the AD to the biologically effective dose (BED), taking into account some of these differences [14]. Assuming that the LQ-model is valid for PRRT, the limits extrapolated from EBRT of 18 Gy and 28 Gy would correspond to BED limits of 32 Gy and 50 Gy,^1^ respectively.

Data from clinical trials of PRRT with ^90^Y-DOTATOC that include dosimetric studies [15, 16] indicate a relationship between renal BED (estimated from pre-treatment scintigraphy or PET) and toxicity. According to MIRD pamphlet no. 20, the BED TD_50/5_ is 44 Gy (α/β = 2.5 Gy), and the threshold for radiation nephropathy is 33 Gy [14]. These results were applied in a prospective clinical trial [17], where 22 patients completed treatment with repeated cycles of ^90^Y-DOTATOC with the aim of not exceeding an accumulated renal BED of 37 Gy (α/β = 2.5 Gy), showing no grade 3-4 toxicity at 18-month follow-up.

When attempting to correlate outcomes to AD or BED, it is essential to use an accurate dosimetric method together with a precise definition of effect. So far, published data on the nephrotoxicity of ^177^Lu-PRRT have mainly been based on planar dosimetry [18, 19], which is known to have inherent limitations regarding the accuracy of activity quantification, and renal function calculated from plasma creatinine as a surrogate for measuring the glomerular filtration rate (GFR). Dose limits similar to those found for EBRT and ^90^Y-DOTATOC have not been determined. This may be because most patients have received the standard of 4x7.4 GBq, which, as shown below, in the majority of patients leads to a renal BED well below the above-mentioned limits, but also because inaccurate dosimetry and renal function estimates may obscure a relationship between dose and toxicity.

Another difficulty encountered when studying the effect of radiation on renal function is the co-existence of other factors that contribute to a decline in GFR. The classic risk factors for nephropathy (diabetes, hypertension, old age, previous nephrotoxic therapies or procedures) have been proposed to increase the risk of renal failure post-PRRT [9]. However, in a subsequent large meta-analysis, the predictive power of these risk factors was questioned [20]. Also, in a recent analysis of patients receiving a kidney dose of on average 19.3 Gy, none of the included risk factors had a significant effect on renal function [18]. The GFR at the start of PRRT could also affect the risk of subsequent renal failure, as suggested by an analysis of 51 patients treated with ^177^Lu-DOTATATE, showing that those who had a reduced initial GFR also received a higher renal AD/administered activity [19]. Renal insufficiency is a late occurring toxicity, and a follow-up time of at least 18 months after treatment is required.

Our group has previously compared the results of different dosimetric methods, including planar and SPECT-based methods, to determine the renal BED after ^177^Lu-DOTATATE treatment [21]. Based on our findings, and in accordance with the indications in MIRD pamphlet no. 26 [22], we designed a Phase II clinical trial using a hybrid planar- and SPECT-based method to continuously evaluate the cumulative renal BED after each cycle, which then guided the number of cycles given. Here, we present the results of an interim analysis of this trial.

The aims of this analysis are to describe how individualised dosimetry-based treatment planning affects the number of cycles each patient receives, and to describe the development of renal function during follow-up, to ensure an acceptable balance between the risks and benefits of therapy. It includes the first 51 patients enrolled in the trial, of 100 patients planned.

## Patients and methods

### Trial design

This is an interim analysis of a Phase II, multicentre, prospective clinical trial using ^177^Lu-DOTATATE to treat metastatic neuroendocrine tumours. The trial is being conducted at two Swedish university hospitals, and has been approved by the regional ethics review board and national regulatory authorities. Further details on the protocol can be found at www.clinicaltrials.gov (NCT01456078). All patients have given their written informed consent to participate.

The primary objective of the trial is to study the efficacy and safety of an individualised dosimetry-based treatment with ^177^Lu-DOTATATE up to a cumulative BED to the kidneys of 27(±2) Gy. Secondary objectives are to study the same aspects but in a selected group of patients without risk factors for renal or haematological toxicity, who may receive treatment up to 40(±2) Gy.

### Study population

The basic eligibility criteria for inclusion are: adult patients with histologically verified, progressive metastatic neuroendocrine tumour with a Ki67 index ≤20%; tumour lesions must be measureable according to RECIST v1.1 and have an uptake on somatostatin receptor scintigraphy that is higher than that of normal liver parenchyma; WHO performance status ≤ 2; normal liver and bone marrow function and a baseline measured GFR ≥50 mL/min/1.73 m^2^, determined by iohexol or ^51^Cr-EDTA clearance. Risk factors for nephropathy are identified at baseline (diabetes, hypertension, age > 70 years, prior liver embolization, and prior chemotherapy).

### Treatment

Each cycle of ^177^Lu-DOTATATE is given at a standard activity of 7.4 GBq at 8-12-week intervals. A reno-protective amino acid infusion is started 30 min before and continued until 8 h after the infusion of ^177^Lu-DOTATATE. The number of cycles received by each patient is determined by the accumulated renal BED, evaluation of renal function, haematological tolerance and trimestral radiological evaluations. All patients are offered treatment up to a BED of 27(±2) Gy (Step 1), as long as there is no progression of the disease or treatment-limiting toxicity. Thereafter, those who have no risk factors for haematological or renal toxicity, non-progressive disease and good tolerance, are offered continued treatment up to a cumulative renal BED of 40(±2) Gy (Step 2).

### Post-therapy imaging and renal dosimetry

After each cycle, four whole-body anterior-posterior planar scintigraphies are performed (at nominal times 1, 24, 48 or 96 and 168 h post-injection). At 24 h post-injection SPECT/CT imaging and an X-ray scout are also acquired. Images are exported in DICOM format and further processed using the LundADose software for 2D/3D dosimetry developed at our department. The procedure for dosimetry is further described in supplemental data, including considerations related to the renal pharmacokinetics. The AD and BED of left and right kidney are calculated separately, and the mean values are determined. For calculation of BED from the time-dose rate curve, a numerical method is used, taking into account repair of sublethal damage during protracted irradiation [23]. A kidney α/β of 2.6 Gy and monoexponential repair with a half-time of 2.8 h are assumed, in analogy with previous publications [9].

### Follow-up of renal function

Renal function is analysed at baseline by serum creatinine, estimation of GFR (eGFR) using the MDRD formula and the measured GFR (mGFR) determined by iohexol or ^51^Cr-EDTA clearance. Renal function is monitored between cycles using serum creatinine and eGFR before each cycle and mGFR is determined at least once a year, after completion of Step 1 and Step 2, and additionally according to investigator criteria. Follow-up time is defined as time from inclusion to point of last follow-up.

For the present analysis, the development of the median mGFR and the annual change in eGFR (ΔeGFR) are determined. The ΔeGFR is calculated for each patient individually by linear regression to eGFR versus time data and dividing the obtained slope by the initial mGFR value (mGFRi).

As an exploratory analysis, the projected time to a clinically significant reduction in renal function (GFR < 30 mL/min/1.73 m^2^) is also included, based on the observation that patients who experience radiation nephropathy tend to exhibit a continued decline in renal function [12]. Thus, as an approximation, the product of mGFRi and ΔeGFR is calculated and the time when the GFR reaches 30 mL/min/1.73 m^2^ (G3 toxicity) determined by extrapolation.

## Results

### Patient population

Inclusion of patients in the trial is ongoing, with 51 patients having been included at the time of analysis. The results presented are in part based on all included patients (median follow-up 18 months, range 1-43), in part based only on the patients who had reached end of treatment (EOT) at the time of analysis (EOT_all_, N = 41, median follow-up 24 months, range 1-43). The patients are further sub-grouped into those who terminated treatment because they had reached the protocol-specified BED limit (EOT_dose_, N = 22, median follow-up 28 months, range 13-39), or due to toxicity, adverse event or progressive disease (EOT_tox/PD_, N = 15) as indicated in the text. Treatment was ongoing in ten patients at the time of analysis, and four were excluded from the analysis of effect and toxicity due to protocol deviations. Five patients had received treatment within Step 2.

The baseline characteristics for all 51 patients are presented in Table 1. Half were women, and the median age at inclusion was 67 years (range 35-80). The most common primary tumour origins were small intestine (73%) and pancreas (10%), the rest being evenly distributed between pulmonary, colorectal and unknown origins. Two thirds of the tumours had a Ki67 index of 0-2% and the remaining one-third 3-20%, at the time of diagnosis. The baseline mGFR (mGFRi) median value was 71 mL/min/1.73 m^2^ (range 48-104), and the median eGFR 87 mL/min/1.73 m^2^ (range 43-159). One third of the patients had mGFRi >80 mL/min/1.73 m^2^ (G0 according to NCI CTCAE v4.0), 49% G1 (60-80 mL/min/1.73 m^2^), and 18% G2 (30-59 mL/min/1.73 m^2^). The prevalence of risk factors for nephropathy at baseline was as follows: none (39%), one (27%), two (25%), and more than two (8%). Two thirds of the patients had a performance status of ECOG 0-1, and one third had ECOG 2.

**Table 1:** Baseline characteristics

Characteristic	Patients	
	No.	%
Sex		
Female	26	51%
Male	25	49%
Age (years)		
Median	67	
Range	35-80	
Primary tumor		
Small intestine	37	73%
Pancreas	5	10%
Lung	3	6%
Colorectal	3	6%
Other/unknown	3	6%
Ki67 index		
0-2%	33	65%
3-20%	18	35%
> 20%	0	0%
Baseline mGFR (mL/min/1.73 m^2^)		
All patients		
Median	71	
Range	48-104	
With risk factors		
Median	69	
Range	48-104	
Without risk factors		
Median	83	
Range	54-96	
Baseline eGFR (mL/min/1.73 m^2^)		
Median	87	
Range	43-159	
Risk factors nephropathy		
0	20	39%
1	14	27%
2	13	25%
> 2	4	8%
Performance status (ECOG)		
0	6	12%
1	27	53%
2	18	35%
3-4	0	0%

### Renal dosimetry

**Fig. 1:**
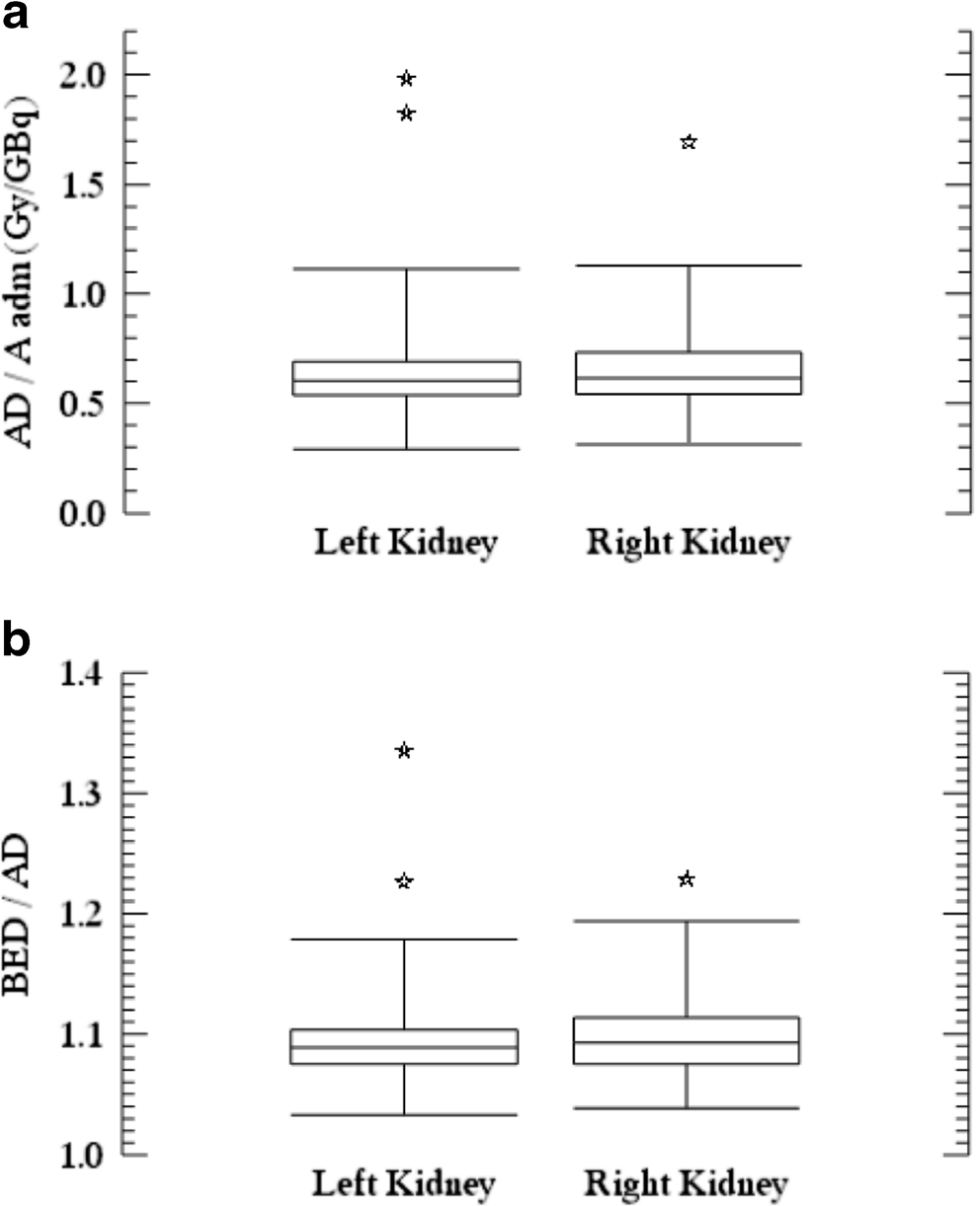
(**a**) Absorbed dose per unit administered activity (Gy/GBq) calculated from a total of 199 cycles. Stars denote outliers. (**b**) BED per absorbed dose. Stars denote the AD/BED from outliers obtained in A

The median AD and BED/cycle for all 51 patients were 4.5 Gy (range 2.2-14.3) and 4.9 Gy (range 2.3-19.1), respectively, calculated from a total of 199 cycles. The wide range was due to both inter- and intrapatient variability. The maximum values were attributable to a patient who was admitted to hospital due to intercurrent obstructive nephrolithiasis a few days after receiving treatment. As shown in Fig. 1, the median AD/administered activity was 0.61 Gy/GBq (range 0.3-1.98), and the median BED/AD was 1.09 (range 1.03-1.34). The median BED/administered activity was 0.67 Gy/GBq (range 0.3-2.6), and the median effective half-life was 51.6 h (range 38-69) (data not shown). The AD and BED of the left and right kidneys were generally similar in the same patient and cycle. The fractional BED deviation, calculated as the difference between the left and right kidney divided by the mean of the left and right kidney, was on average -1.8% for individual cycles and -0.2% for the whole treatment. No systematic difference in the BED of the left and right kidneys was seen, although in the above-mentioned patient with obstructive nephrolithiasis the BED differed for non-methodological reasons.

**Fig. 2:**
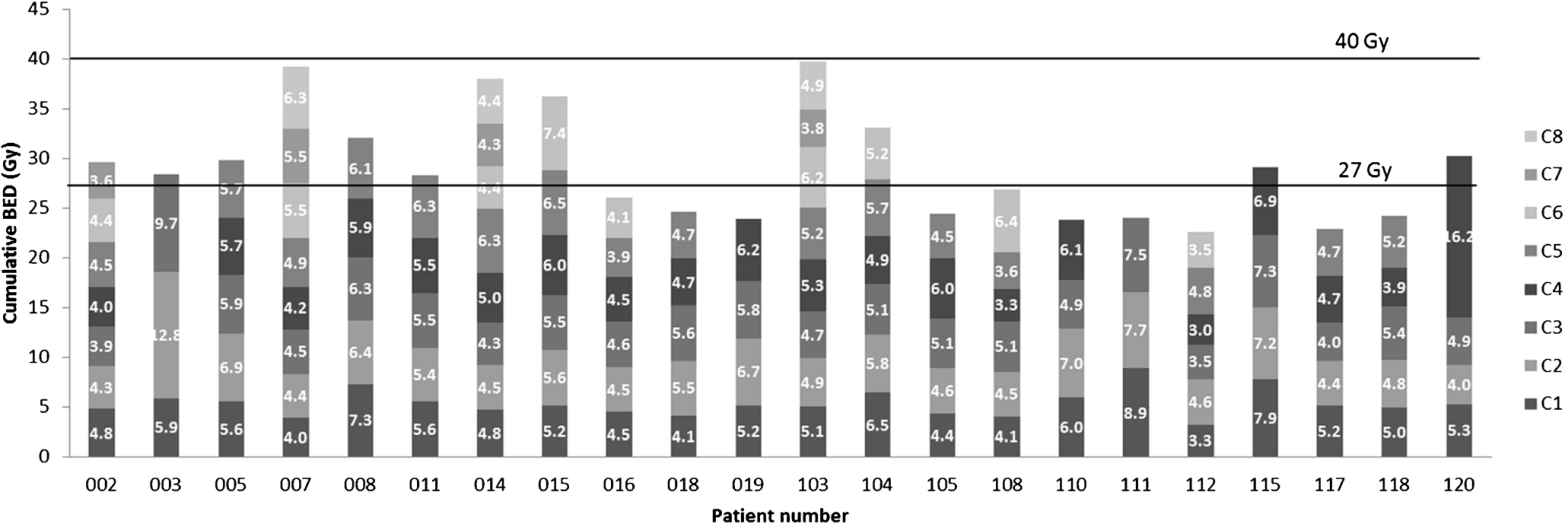
Cumulative BED, number of treatment cycles received and BED/cycle for the patients who completed treatment after reaching the protocol-specified BED limit (EOT_dose_)

The median interval between cycles was 77 days (range 56-210). The median number of cycles received within Step 1 by the EOT_dose_ patients was five (range 3-7) reaching a mean renal BED of 26.5 Gy (range: 22.6-32.1). The five patients who completed treatment within Step 2 received a median of seven cycles (range 5-8) reaching a mean renal BED of 37.3 Gy (range: 33.1-39.8). The individual results are illustrated in Fig. 2, where the considerable inter- and intrapatient variability regarding the BED/cycle and the total number of cycles is evident. As an illustrative example, we can compare patients 111 and 112, who both received treatment up to an accumulated BED of approximately 25 Gy, one after only three cycles, while the other required six cycles. Conversely, patients 015 and 016 received the same number of cycles, but very different total kidney BEDs (36 Gy vs. 26 Gy).

**Fig. 3:**
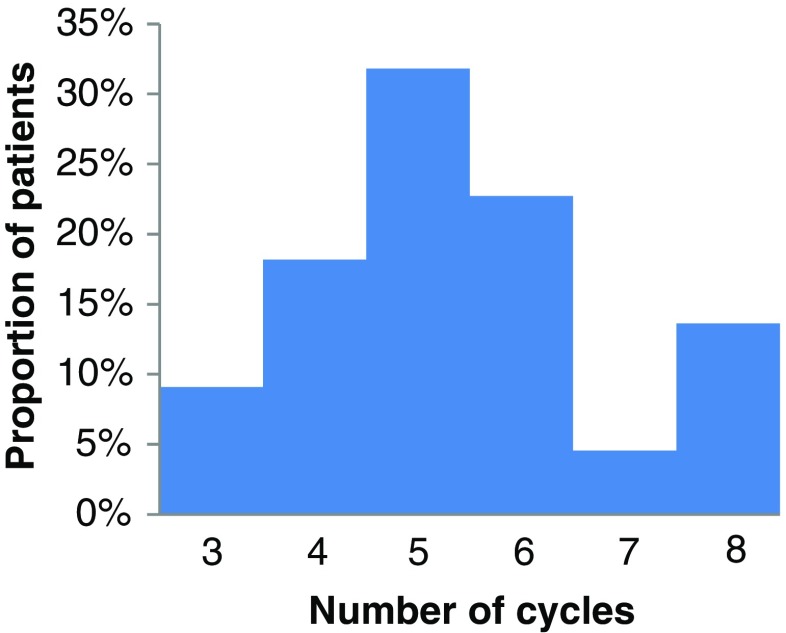
Frequency distribution of number of cycles delivered within the protocol-specified BED-limits

In the EOT_dose_ group 16/22 patients (73%) received more and 2/22 (9%) received less than four cycles. As illustrated in Fig. 3, this leaves only four patients (18%) for whom four is the optimal number of cycles according to the BED limits used in this protocol. If all 22 patients had received four cycles, the mean renal BED would have been 22.7 (range 14.3-38.1) Gy (assuming the same BED in cycle 4 as in cycle 3 for the patients who only received three cycles).

### Renal function vs. cumulative renal BED

**Fig. 4:**
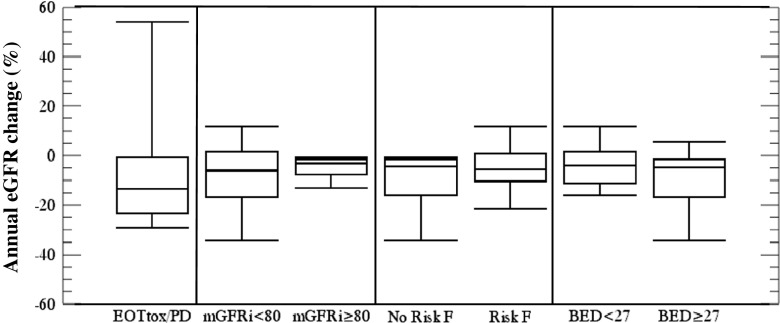
Pair-wise comparisons of ΔeGFR for the EOT_dose_ group (N = 22) and an eGFR follow-up >6 months categorized by initial mGFR (mL/min/1.73 m^2^), the existence or not of risk factors (Risk F) for nephrotoxicity, and the cumulative renal BED (Gy). The results for the EOT_tox/PD_ group (N = 10) are presented in the column to the left

In the EOT_all_ group, data on eGFR development ≥ 6 months from baseline were available for 32 patients (median follow-up 19 months), while follow-up data on mGFR were available for 22 (median follow-up 22 months). The contribution of cumulative BED, mGFRi, and pre-existing risk factors for nephrotoxicity was compared pair-wise (Fig. 4). Based on the observation that the EOT_tox/PD_ patients were overrepresented in the group exhibiting a more rapid decline in GFR (see Table 2), these were analysed separately. The patients with the smallest GFR-loss were those with an mGFRi ≥ 80 mL/min/1.73 m^2^, including those treated to a BED of 40(±2) Gy, and those with the largest GFR loss was the EOT_tox/PD_ group. All patients with a GFR loss > 10% either had mGFRi < 80 mL/min/1.73 m^2^ and/or risk factors for nephrotoxicity. The dose-limiting toxicity was haematological (Table 2).

**Table 2:** Summary of key patient-specific data for the EOT_all_ population (N = 41)

Patient	# of cycles	Risk factors for renal tox	Cumulative renal AD (Gy)	Cumulative renal BED (Gy)	Cumulative whole-body AD (Gy)	Baseline mGFR (mL/min/1.73 m^2^)	∆eGFR (%)	Follow-up (months)	Reason for EOT
A									
112	6	Y	21	23	1.5	94	−7.6	28	Dose
117	5	Y	21	23	N/A	66	−10.5	18	Dose
019	4	Y	22	24	1.4	71	1.0	18	Dose
105	5	Y	23	24	2.4	85	−1.6	17	Dose
110	4	Y	22	24	1.7	63	−1.3	32	Dose
111	3	Y	21	24	0.9	68	3.2	28	Dose
118	5	Y	22	24	1.7	70	−6.2	16	Dose
018	5	Y	23	25	1.9	59	11.5	20	Dose
016	6	N^1^	24	26	1.8	60	−16	22	Dose
108	6	Y	25	27	2.7	97	−13.2	28	Dose
003	3	N^2^	24	28	2.0	78	−34.2	19	Dose
011	5	Y	25	28	1.6	72	−2.1	30	Dose
115	4	Y	26	29	1.2	61	5.6	21	Dose
002	7	Y	27	30	3.0	62	−5.5	39	Dose
005	5	Y	27	30	2.0	69	−9.1	39	Dose
120	4	Y	26	30	1.5	78	−16.2	13	Dose
008	5	Y	29	32	1.6	48	−19.5	32	Dose
104	6	N	30	33	1.2	83	−4.3	29	Dose
015	6	N	32	36	2.1	82	−1.7	25	Dose
014	8	N	35	38	2.2	88	−1.5	28	Dose
007	8	N	36	39	3.9	85	−7.4	35	Dose
103	8	N	37	40	1.6	84	−0.7	37	Dose
Median	5		25	28	1.7	72	−4.9	28	
B									
001	4	N	18	20	1.3	87	3.4	8	PD
017	5	Y	23	25	1.8	71	−29.1	15	PD
124	3	Y	14	15	0.9	67	54	6	PD
010	2	Y	6.9	7.4	0.9	73	−7.8	7	SAE
004	2	N	9.1	9.8	N/A	57	−2.4	43	Hem tox
101	4	Y	16	18	3.5	62	−7.3	9	Hem tox
106	2	N	9.2	10	0.7	96	−27.4	24	Hem tox
107	3	N	12	12	1.8	91	−18.5	34	Hem tox
119	3	N	15	17	0.9	73	−21.8	17	Hem tox
122	2	N	8	8.6	1.0	56	−21.2	13	Hem tox
Median	3		13	14	1.0	72	−13.1	15	
C									
009	1	Y	3.9	4.2	0.8	54	N/A	5	Death/PD
012	1	Y	4.3	4.7	0.4	52	N/A	2	Death/PD
114	1	Y	3.8	4.2	0.6	104	N/A	1	Death^3^
013	5	N	34	38	3.2	56	N/A	29	Prot dev
109	4	N	26	28	N/A	53	N/A	33	Prot dev
113	6	Y	26	28	N/A	75	N/A	3	Prot dev
116	5	Y	20	21	N/A	69	N/A	9	Prot dev
102	2	Y	8.1	8.7	1.9	73	N/A	5	Hem tox
Median	2		13	15	1.25	54	N/A	4	

Data are grouped according to reason for EOT: (A) EOT_dose_, (B) EOT_tox/PD_, (C) Prot dev and/or follow-up <6 months. ∆eGFR (annual eGFR-change) was calculated as the slope of a line fitted to eGFR-versus-time data, divided by the initial mGFR. AD absorbed dose, BED biologically effective dose, mGFR measured glomerular filtration rate, eGFR estimated glomerular filtration rate, EOT end of treatment, PD progressive disease, Prot dev protocol deviation, Hem tox haematological toxicity, SAE serious adverse event. Footnotes 1: not eligible for step 2 due to previous chemotherapy, 2: intercurrent obstructive nephrolithiasis, 3: cardiac arrest in the context of a sepsis.

### Renal function over time

**Fig. 5:**
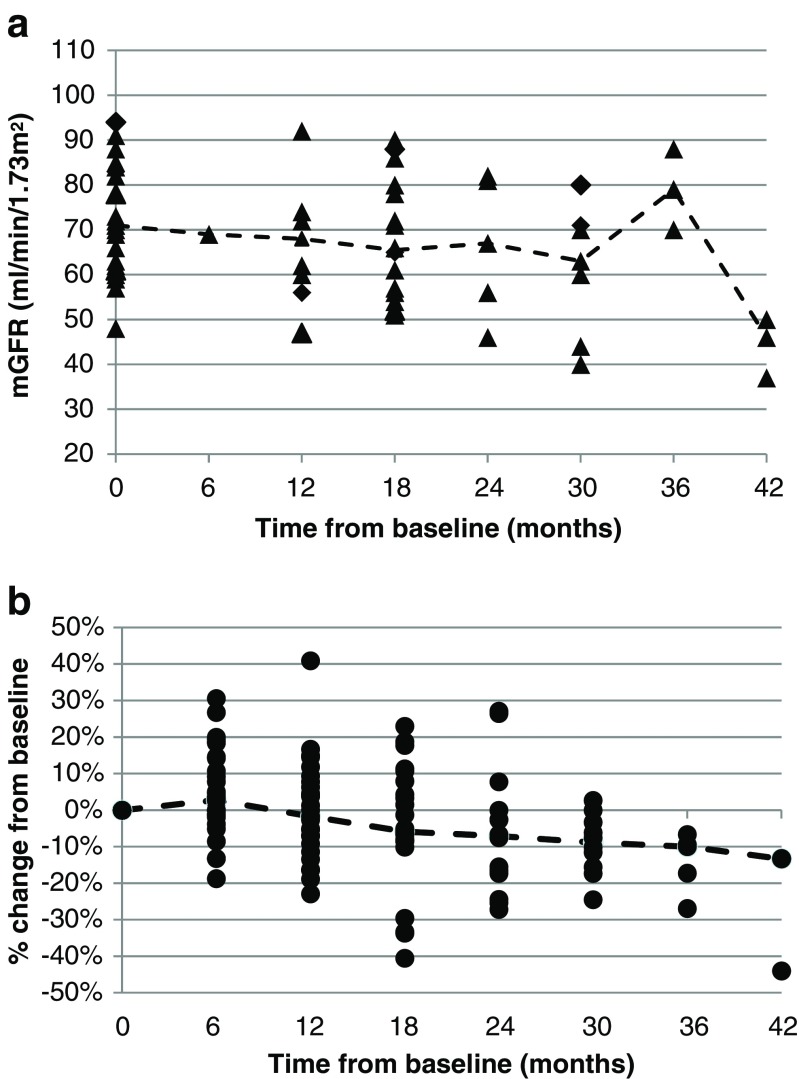
Development of renal function over time (EOT_all_). (**a**) Absolute values of mGFR and median mGFR (dotted line) (**b**) % eGFR change from baseline and median % eGFR change (dotted line)

As illustrated in Fig. 5a, median mGFR decreased with increasing follow-up, although the number of patients with a follow-up >24 months is small, making it difficult to draw any conclusions beyond this point in time. The mean absolute annual change in mGFR was -4.3 mL/min/1.73 m^2^ (range +11 to -30), while the mean annual GFR-loss in a normal population of a similar age range is between 0.4 ± 3.6 and 1.8 ± 2.6 mL/min [24]. The loss of renal function among the PRRT-treated patients was thus slightly more rapid, although no grade 3-4 toxicity (i.e. GFR < 30 mL/min/1.73 m^2^) was observed. As illustrated in Fig. 5b, four patients exhibited a decrease in eGFR >30% compared to baseline, one of whom was the patient with obstructive nephrolithiasis, two had progressive disease at the time the decrease in renal function was confirmed, and in the fourth patient the decline in eGFR improved, from -30% to -24%, with further follow-up.

Among the five patients who received treatment within Step 2, the mean change in mGFR from baseline to the last point of follow-up was -7% (range -14% to +5%), while the mean change in eGFR was -14% (range -25% to +7%). Two patients exhibited a reduction in renal function from G0 to G1, while G0 renal function was maintained in three patients throughout follow-up (data not shown). The median mGFR follow-up for this subgroup was 29 months (range 24-37).

**Fig. 6:**
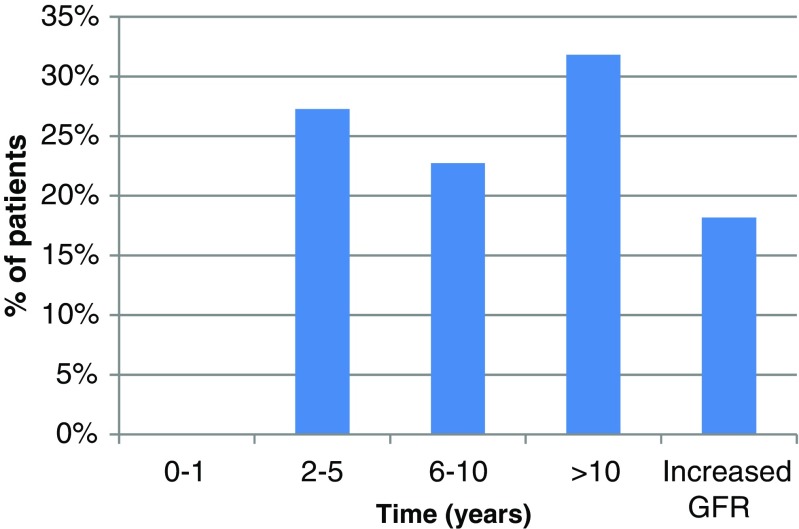
Projected time to significant reduction in GFR (<30 mL/min/1.73 m^2^) in the EOT_dose_ group, assuming a continued and constant rate of decline in renal function

Figure 6 shows results of the exploratory analysis of the time when significant renal toxicity would be reached (grade 3, GFR <30 mL/min/1.73 m^2^). Six patients (19%) actually showed an improvement in GFR during follow-up, five of which had risk factors for nephrotoxicity. None had a projected decline to GFR < 30 mL/min/1.73 m^2^ within 1 year, but it was projected within 2-5 years for six patients (34%). The projected time to GFR <30 mL/min/1.73 m^2^ was 6-10 years in five patients (22%) and longer than 10 years in seven patients (25%).

## Discussion

In the present trial, we have prospectively collected data on renal function and dosimetry after ^177^Lu-DOTATATE PRRT and individualised treatment based on an assumed maximum tolerable dose to risk organs, with the aim of maximising the AD to tumour tissue and thus the probability of antitumor effect. When seeking to define a relationship between absorbed dose and toxicity, it is essential to have accurate measurements of both. In this trial, we used an ambitious dosimetric method together with regular measurement *and* estimation of renal function, to be able to draw firm conclusions as the number of patients and follow-up time increases.

A challenge when designing the trial was to define the BED limits in a way that increased the likelihood of therapeutic gain without venturing patient safety. As summarised in the introduction, the evidence available would indicate a threshold for long-term nephrotoxicity of 32-33 Gy, without taking into account clinical risk factors. Based on Bodei et al [9] risk factors for nephrotoxicity seemed to clearly affect the recommendable BED limits. Based on this, we defined the renal BED-limits to 27 for patients with risk factors and 40 Gy for those without.

When the physician sees a patient after X cycles and a cumulated renal BED of Y Gy, aware that the renal dose varies from one cycle to another, he/she has to attempt to predict the cumulative BED with additional cycles. Certain variability in the final dose (±2 Gy) was, therefore, added as a compromise with clinical reality. In a previous study we also investigated the combined uncertainties in SPECT/CT-based renal dosimetry in 177Lu-PRRT, using the same SPECT/CT methods as in this study, and were able to quantify one standard deviation to 6% [25]. Currently we are performing uncertainty analyses for the hybrid planar-SPECT method used in this trial. The results are beyond the scope of this work and will, therefore, be presented separately.

One may object to there being no upper dose limit for the bone marrow (BM) in this trial. Sandstrom *et al*, however, found that the BM AD/cycle was <0.2 Gy and concluded that it was the renal AD that was dose-limiting in 98% of the patients, even when permitting a renal AD up to 29 Gy [11]. The acute BM toxicity is monitored using blood sampling at regular intervals during treatment. Since acute BM toxicity is easily detected, and the limit of BM AD is yet undefined for PRRT, we decided to study BM AD in an exploratory substudy, the results of which will be presented separately. For a majority of the patients the whole-body AD has been determined (see Table 2).

A fundamental difficulty with dosimetry in PRRT is the lack of radiobiological data regarding dose-response relationships for tumour and normal tissues. The data that does exist suffers from considerable variability in radionuclides, dosimetric methods, and clinical data making pooling of results difficult. We are obliged to extrapolate from EBRT and the LQ-model without knowing its applicability to PRRT when taking into account the numerous physical and biological differences between the treatment modalities. To be able to go forward with studies incorporating rigorous dosimetry and clinical follow-up which may add to the knowledge base, we inevitably have to make certain assumptions a priori based on the best available evidence, and continuously re-evaluate the correctness of these assumptions as results become available.

Estimation of GFR based on plasma creatinine is subject to inaccuracies which vary depending on the method used. The MDRD and the Cockcroft-Gault formulas are the most commonly used, and recent reviews support MDRD as being somewhat more reliable [26, 27]. Superior to any estimation of GFR is its measurement, limited on the other hand by its more invasive nature. In this trial we followed renal function using both estimated and measured values. We used the less invasive method (eGFR) for close follow-up of changes in renal function, with regular mGFR measurements to calibrate the eGFR values. Assuming that the difference between eGFR and mGFR data is constant over time for the same patient, the possible bias in eGFR values will not affect the rate of the change. The percent change on the other hand depends on the value used for normalization, for which the initial mGFR value was used.

In our patient material renal function gradually declined after treatment with ^177^Lu-DOTATATE, at a rate that seemed slightly higher than what could be expected in a corresponding normal population. The decline was moderate, with no grade 3-4 toxicity observed so far. Patients with risk factors for nephrotoxicity, or a moderately reduced mGFRi, seemed at risk of a more rapid decline in renal function than their healthier counterparts. A possible explanation is that risk factor patients have a lower number of functioning nephrons at baseline than their no-risk factor counterparts and are, therefore, at greater risk of suffering a clinically significant reduction in GFR as a result of nephrotoxic therapies such as PRRT. The patients showing the highest risk of GFR decline were actually those who were not able to complete PRRT as planned, due to non-renal toxicity/adverse event or progressive disease. This suggests that factors other than the radiation dose to the kidneys caused their nephropathy. Our results support the dose limit proposed in this trial for high-risk patients (27 ± 2 Gy), while indicating that low-risk patients may well tolerate further treatment.

The primary objective of PRRT in patients with progressive NETs is to bring the tumour under control. Some deterioration in renal function is acceptable, as long as it does not affect the patients’ quality of life for the remainder of their lifetime. Patients with radiation nephropathy tend to have a continuous progressive decline in renal function. Although the rate of this decline is unknown, it is valuable from the clinician’s point of view to have some idea of the prognosis for toxic effects in relation to the expected therapeutic effects of the treatment and survival of the patient. For this reason we have attempted to predict the long-term effects on renal function by extrapolating the rate of change of eGFR calculated for each patient to give an approximate time to significant renal insufficiency, defined as ≥ G3. As for any exploratory analysis, the results have to be interpreted with caution.

Our findings clearly show that individualising treatment based on patient selection and detailed dosimetry results in considerable variability in the number of cycles each patient is able to receive within the pre-specified BED limits. Not only does the AD/cycle vary between patients, but also between cycles in the same patient. The reason for the intra-individual variations is presently unclear, although it may be that blood pressure and degree of hydration, and thus renal perfusion, varied between cycles secondarily affecting the residence time in the kidney and thereby the AD. Previous authors [18] have stated that the risk of nephrotoxicity after standard PRRT with four cycles is minimal, rendering dosimetry superfluous. However, if the goal is the optimization of efficacy vs. toxicity, or if retreatment is being considered, we maintain that dosimetry is necessary, based on the findings in the present analysis.

## Conclusions

Individualised dosimetry-based PRRT is feasible and safe, with the BED limits used in this protocol, with the caveat of the limited follow-up inherent to an interim analysis. The trial will continue as planned.

## Electronic supplementary material

Below is the link to the electronic supplementary material.

ESM 1 (DOCX 35 kb)

## Footnotes

1 If not otherwise specified, an α/β value of 2.6 Gy has been used for the BED-calculations.
