# Supplementary material for: Individualised 177Lu-DOTATATE treatment of neuroendocrine tumours based on kidney dosimetry
Source: Eur J Nucl Med Mol Imaging. 2017 Mar 22;44(9):1480–9. doi: 10.1007/s00259-017-3678-4 (PMC5506097; doi:10.1007/s00259-017-3678-4)
Supplement: Supplementary file 1 — (DOCX 35 kb) [file 259_2017_3678_MOESM1_ESM.docx]

# Supplemental data

All dosimetric calculations were made using the in-house program package LundADose, and parts of the protocol for imaging and dosimetry are described in the MIRD 26 pamphlet, as the patient example entitled “Lund”.

## Imaging and calibration

In all acquisitions medium-energy general purpose collimators were used with an energy window centred at 208 keV and with a width of either 15% or 20%, depending on the energy resolution of the camera system used. Further details on the imaging protocol are specified in table S1.

| Image type | Matrix size (pixels) | Pixel size (mm) | Acquisition time |
| --- | --- | --- | --- |
| Whole-body 1h | 1024x256 | 2.2 x 2.2 | 10 min |
| Whole-body 24, 48/96 and 168h | 1024x256 | 2.2 x 2.2 | 20 min |
| SPECT/CT 24 h | 128x128 | 4.4 x 4.4 | 60 projections x 45s |

**Table S1.** Specifications regarding the image acquisitions

Since the trial was conducted at two sites using different camera systems, a common calibration method was used to ensure comparable data from both. The calibration factor used for converting count rate to activity, for a given camera system, was identical for planar whole-body images and SPECT. The calibration factor was derived from a planar scan, of a thin layer of ^177^Lu in a Petri dish placed in air. The dose calibrators were calibrated using a ^177^Lu source with activity statement traceable to a standard laboratory. An exchange of data between the two sites was also performed, and the results of these comparison studies will be reported in a separate work.

## Image processing

For SPECT image reconstruction an iterative OSEM algorithm was used with 8 iterations and 10 subsets, and including corrections for CT-based attenuation, scatter (ESSE method), and collimator response. Planar whole-body images were quantified on a pixel-by-pixel basis, using the conjugate-view method. For this purpose the X-ray scout image was first converted to an attenuation map for 208 keV, while scatter correction was performed using a Wiener filter based on depth-dependent Monte Carlo calculated scatter kernels. All whole-body images were registered to the scout image using non-rigid registration methods. The quantified SPECT study was used as input to a voxel-based Monte Carlo calculation (EGS4) of the absorbed dose-rate at 24 hours.

Regions and volumes of interest (ROIs and VOIs) were delineated in the planar and SPECT/CT images, respectively, by specially trained technologists. For planar images, ROIs over both kidneys were delineated, together with a background ROI just below each kidney for background correction. As the purpose of the planar-derived values was to capture the kinetics rather than the total kidney activity, the kidney ROIs were generally drawn well within the kidney border, and were always placed in the cortex-medulla region. VOIs for analysis of the absorbed-dose rate images were delineated mainly based on the CT, and encompassed the cortex and medulla regions of the left and right kidneys.

## Dosimetry

The time-activity data from the planar images were combined with the absorbed-dose rate value at 24 h, derived from the SPECT/CT image, thus generating a set of time dose-rate values. A curve was fitted to this data set, and by analytical integration from the curve parameters the renal AD for each kidney was determined as the area under the curve. The BED was also calculated by numerical convolution between the absorbed dose-rate curve and a mono-exponential repair function, using an α/β of 2.6 Gy and a repair half-time of 2.8 h.

For the curve shape we adopted a mono-exponential curve fitted to data from 24 h and onwards, combined with a straight line determined from the 1 h data point and the value of the exponential curve at 24 h. The reason for applying this trapezoid integration for the early phase was that, with the current patient acquisition protocol, we found that data do not support more elaborate analyses. The underlying renal pharmacokinetics is bi-phasic, with an early peak related to an initial fast phase of extravasation and excretion of ^177^Lu-Dotatate, followed by a slower phase of renal uptake and washout [1, 2]. In view of the findings in our data and by others, that 70%-75% of the absorbed dose delivered during one cycle is due to the area under the curve after approximately 24 h, the curve-fitting contribution to the overall absorbed-dose uncertainty will to a large extent be governed by the estimation of the pharmacokinetics of the slow phase [3, 4]. According to [3] a data point acquired later than 100 h is advised for ^177^Lu-peptides, which was well fulfilled in our protocol where the last data point was at 168 h.

1. Delker A, Ilhan H, Zach C, Brosch J, Gildehaus FJ, Lehner S et al. The Influence of Early Measurements Onto the Estimated Kidney Dose in [(177)Lu][DOTA(0),Tyr(3)]Octreotate Peptide Receptor Radiotherapy of Neuroendocrine Tumors. Mol Imaging Biol. 2015;17(5):726-34. doi:10.1007/s11307-015-0839-3.

2. Brolin G, Gustafsson J, Ljungberg M, Gleisner KS. Pharmacokinetic digital phantoms for accuracy assessment of image-based dosimetry in (177)Lu-DOTATATE peptide receptor radionuclide therapy. Physics in medicine and biology. 2015;60(15):6131-49. doi:10.1088/0031-9155/60/15/6131.

3. Guerriero F, Ferrari ME, Botta F, Fioroni F, Grassi E, Versari A et al. Kidney dosimetry in (1)(7)(7)Lu and (9)(0)Y peptide receptor radionuclide therapy: influence of image timing, time-activity integration method, and risk factors. Biomed Res Int. 2013;2013:935351. doi:10.1155/2013/935351.

4. Gustafsson J, Nilsson P, Gleisner KS. On the biologically effective dose (BED)-using convolution for calculating the effects of repair: II. Numerical considerations. Physics in medicine and biology. 2013;58(5):1529-48. doi:10.1088/0031-9155/58/5/1529.
